# Ectopic expression of human airway trypsin‐like protease 4 in acute myeloid leukemia promotes cancer cell invasion and tumor growth

**DOI:** 10.1002/cam4.2074

**Published:** 2019-03-07

**Authors:** Ruhong Yan, Meng Liu, Yae Hu, Lina Wang, Can Wang, Yizhi Jiang, Quansheng Zhou, Xiaofei Qi, Ningzheng Dong, Qingyu Wu

**Affiliations:** ^1^ Cyrus Tang Hematology Center, Collaborative Innovation Center of Hematology, State Key Laboratory of Radiation Medicine and Prevention Soochow University Suzhou China; ^2^ Department of Clinical Laboratory The Affiliated Suzhou Hospital of Nanjing Medical University Suzhou China; ^3^ MOH Key Laboratory of Thrombosis and Hemostasis Jiangsu Institute of Hematology Suzhou China; ^4^ Department of Urology of the First Affiliated Hospital of Soochow University Suzhou China; ^5^ Jiangsu Key Laboratory of Preventive and Translational Medicine of Geriatric Disease Suzhou China; ^6^ Cardiovascular and Metabolic Sciences Cleveland Clinic Cleveland Ohio

**Keywords:** Acute myeloid leukemia (AML), cancer progression, human airway trypsin‐like protease 4 (HAT‐L4), matrix metalloproteinase (MMP), type II transmembrane serine protease (TTSP)

## Abstract

Transmembrane serine proteases have been implicated in the development and progression of solid and hematological cancers. Human airway trypsin‐like protease 4 (HAT‐L4) is a transmembrane serine protease expressed in epithelial cells and exocrine glands. In the skin, HAT‐L4 is important for normal epidermal barrier function. Here, we report an unexpected finding of ectopic HAT‐L4 expression in neutrophils and monocytes from acute myeloid leukemia (AML) patients. Such expression was not detected in bone marrow cells from normal individuals or patients with chronic myeloid leukemia, acute lymphocytic leukemia and chronic lymphocytic leukemia. In AML patients who underwent chemotherapy, persistent HAT‐L4 expression in bone marrow cells was associated with minimal residual disease and poor prognostic outcomes. In culture, silencing HAT‐L4 expression in AML–derived THP‐1 cells by short hairpin RNAs inhibited matrix metalloproteinase‐2 activation and Matrigel invasion. In mouse xenograft models, inhibition of HAT‐L4 expression reduced the proliferation and growth of THP‐1 cell–derived tumors. Our results indicate that ectopic HAT‐L4 expression is a pathological mechanism in AML and that HAT‐L4 may be used as a cell surface marker for AML blast detection and targeting.

## 1 INTRODUCTION

Type II transmembrane serine proteases (TTSPs) are a group of trypsin–like enzymes with common modular features: an N‐terminal transmembrane domain and a C‐terminal serine protease domain.^1^, ^2^ TTSPs act on the cell surface in many tissues to regulate physiological functions such as iron absorption, salt–water balance, food digestion and epidermal integrity.^3^, ^4^ Defects in TTSPs can cause major health problems, such as iron deficiency anemia,^5^, ^6^ hypertension,^7^, ^8^, ^9^ heart failure,^10^, ^11^, ^12^, ^13^, ^14^ malnutrition,^15^, ^16^ and skin disorders.^17^, ^18^, ^19^, ^20^

In cancers, TTSP overexpression has been shown to enhance oncogenic signaling and promote tumor progression.^21^, ^22^ Most TTSP overexpression occurs in solid tumors. For example, increased levels of hepsin, matriptase and TMPRSS2 have been found in prostate, breast, kidney and ovarian cancers.^21^, ^22^, ^23^, ^24^, ^25^ More recently, ectopic matriptase expression was reported in B‐cell lymphoma and chronic lymphocytic leukemia (CLL),^26^, ^27^ indicating that abnormal TTSP expression and/or activities may be involved in malignant hematological disorders.

Human airway trypsin‐like protease 4 (HAT‐L4) is a TTSP of 48 kDa, consisting of an N‐terminal cytoplasmic tail, a transmembrane domain, a SEA (sea urchin sperm protein/enteropeptidase/agrin) domain and a C‐terminal protease domain.^1^ HAT‐L4 is expressed in epithelial cells and exocrine glands in many tissues including the skin, esophagus, trachea, tongue, bladder and uterus.^28^, ^29^ In mice, HAT‐L4 is dispensable for embryonic development and postnatal hematopoiesis.^29^ In high temperatures, HAT‐L4–deficient newborn pups were prone to body fluid loss, indicating that HAT‐L4 is important for normal epidermal barrier function.^29^

Here, we report an unexpected finding of ectopic HAT‐L4 expression in bone marrow cells from patients with acute myeloid leukemia (AML), the most common type of acute leukemia in adults.^30^, ^31^ AML is caused by chromosomal translocations and mutations in the genes regulating hematopoietic cell growth and differentiation, leading to rapid clonal growth of abnormally differentiated myeloid precursors in the bone marrow.^30^, ^31^ The abnormal HAT‐L4 expression was not detected in bone marrow cells from patients with other types of leukemia, including chronic myeloid leukemia (CML), acute lymphocytic leukemia (ALL) and CLL. To understand the significance of HAT‐L4 expression in AML, we conducted functional studies in cells and mouse xenograft models. Our results show that ectopic HAT‐L4 expression promoted AML cell invasion in vitro and tumor growth in vivo.

## 2 MATERIALS AND METHODS

### 2.1 Patient samples

This study was approved by the ethics committee of the Soochow University and conducted in accordance with the Declaration of Helsinki. The study included 21 healthy volunteers and 165 leukemia patients (60 first‐visit and 105 post treatment). Leukemia classification was based on the French‐American‐British (FAB) guidelines.^32^ All of the participants gave written informed consent. Peripheral blood and bone marrows were drawn into tubes with anticoagulant. Red blood cells were lysed with buffer containing 155 mM NH_4_Cl, 12 mM NaHCO_3_ and 0.1 mM EDTA. White blood cells were isolated for flow cytometric or mRNA and protein analyses.

### 2.2 Cell culture

Hematological cancer cells (U937, HL‐60, NB4, HEL, SHI‐1, THP‐1, SH‐2, KU‐812, MEG‐01, K562, Namalwa, Raji, MOLT4, CCRF‐CEM, Jurkat, RPMI‐8226 and U226) and HeLa cells were cultured in RPMI 1640 medium (Hyclone) with 10% fetal bovine serum (FBS), as described previously.^27^ Hs 505.T and Chinese hamster ovary (CHO) cells were cultured in Dulbecco's Modified Eagle's medium (DMEM) (Hyclone) with 10% FBS. The cell lines were maintained in our institute and not authenticated by STR profiling.

### 2.3 Quantitative RT‐PCR

Total RNAs extracted from cells using TRIzol reagents (Ambion) were used to make cDNAs with reverse transcriptase (Thermo Scientific). Quantitative (q) PCR was done using the StepOne system with the SYBR Green PCR Master Mix (Applied Biosystems). The primers used to amplify human HAT‐L4 cDNA fragments were HAT‐L4‐F (5’‐CCC GCA GTG AAA CGA AAT G‐3’) and HAT‐L4‐R (5’‐TCT GGC TTG CCG AAG TGT A‐3’). The primers used to amplify human MMP‐2 cDNA fragments were hMMP‐2 F (5’‐CCC CAA AAC GGA CAA AGA G‐3’) and hMMP‐2 R (5’‐CTT CAG CAC AAA CAG GTT GC‐3’). Human GAPDH or β‐actin cDNAs were used as controls.

### 2.4 RNA interference

Short hairpin RNAs (shRNAs) targeting the human *TMPRSS11f* gene, encoding HAT‐L4, and scrambled shRNAs were synthesized (GenePharma, Shanghai, China). In addition, shRNAs targeting the human *MMP‐2* gene (shMMP‐2) and scrambled shRNAs (shNC) were synthesized (GenePharma). Lentiviruses containing the shRNAs were transduced into cultured THP‐1 cells. After 12 hours, the medium was replaced by RPMI 1640. The cells were collected after 72 hours and analyzed using flow cytometry for transduction efficiency. qRT‐PCR was used to analyze HAT‐L4 and MMP‐2 mRNA levels to identify shRNAs with the best silencing efficiency. Sequences of *the TMPRSS11f gene* targeted by the selected shRNAs are shown in Figure S1. Sequences for MMP‐2 knocking down shRNAs are TTCTCCGAACGTGTCACGT (shNC) and GCGAGTGGATGCCGCCTTTAA (shMMP‐2).

### 2.5 Plasmid constructs

The plasmid expressing human HAT‐L4 was described previously.^29^ Plasmids expressing HAT‐L4 mutants (R and R1) resistant to shRNA targeting (Figure S1) were made by site–directed mutagenesis. Recombinant HAT‐L4 proteins contained a C‐terminal V5 tag that allowed detection by an anti‐V5 antibody (Invitrogen) in Western blotting.^33^

### 2.6 Western blotting

Cultured or blood– and bone marrow–derived cells were lysed in a solution containing 1% (v/v) Triton X‐100.^34^ Proteins in the lysate were quantified using a BCA‐100 Protein Quantitative kit (Thermo Scientific) and analyzed (10 µg per lane) using SDS‐PAGE and Western blotting using an antibody against human HAT‐L4 (2.7 μg/mL; made in our laboratory^29^) and a horseradish peroxidase (HRP)–conjugated secondary antibody (0.2 μg/mL, Bioworld, BS13276). An anti‐GAPDH antibody (50 ng/mL, GenScript, A00192) was used in controls.

### 2.7 Flow cytometry

Cells were stained with antibodies conjugated with fluorescein isothiocyanate (FITC), phycoerythrin (PE), or peridinin‐chlorophyll‐protein complex (PerCP), as described previously.^35^ Briefly, the cells (in 100 µL buffer) were incubated at room temperature for 30 minutes with the conjugated‐antibodies against HAT‐L4 (described above), leukocytes (CD13‐PE; 347837), monocytes (CD14‐PE; 347464) or lymphocytes (CD19‐PE; 349209) (all from BD Biosciences). Isotype–matched and conjugated IgGs (IgG1‐FITC, 551954; IgG1‐PE, 555749; IgG1‐PerCP, 559425, BD Biosciences) were used as negative controls. Data acquisition and analysis were done using the FACSCalibur system (BD Biosciences) and FlowJo software (Tree Star).

### 2.8 MMP‐2 assay

Matrix metalloproteinase‐2 (MMP‐2) activity was examined with a fluorimetric assay (SensoLyte 520, AnaSpec).^36^ The conditioned media from HAT‐L4–expressing CHO and control cells with or without recombinant pro‐MMP‐2 (902‐MP‐010, R&D Systems) were incubated with a fluoro‐peptide at 37°C over time. The fluorescence intensity was monitored at excitation and emission wavelengths of 485 and 535 nm, respectively, in a plate reader (SpectraMax M5, Molecular Devices).

### 2.9 Immunofluorescent staining

Cells were fixed with 4% paraformaldehyde, pretreated with 5% bovine serum albumin (BSA) for 1 hour and stained with anti‐HAT‐L4‐FITC and anti‐CD13‐PE (BD Biosciences, 347837) antibodies at room temperature for 30 minutes. The cells were placed on coverslips and mounted with a DAPI solution (Fluoromount‐G, Southern Biotech) to stain cell nuclei. The slides were examined with a confocal microscope (FV1000, Olympus), as described previously.^9^

### 2.10 Cell proliferation assay

THP‐1 cells were transduced with scrambled shRNAs (shNC cells) and HAT‐L4 targeting shRNAs (shH cells). As another control for shRNA‐targeting specificity, THP‐1 cells were transduced with the HAT‐L4–targeting shRNAs and mutant HAT‐L4 cDNAs in which corresponding shRNA–targeting sites were mutated (shR cells) (Figure S1). The cells were cultured in 96‐well plates (1×10^5^ cells/well) in RPMI 1640 medium at 37°C. Cell proliferation was analyzed with a Cell Counting Kit‐8 assay (CCK‐8, Beyotime Biotechnology).

### 2.11 Cell migration and invasion assays

Transwell assays (BD Biosciences) were used to test cell migration and invasion.^27^ The outside bottom of the top chamber was coated with fibronectin (Sigma‐Aldrich). For the migration assay, the cells (2 × 10^5^) were added into the upper chamber in serum–free RPMI 1640. For the invasion assay, the inside bottom of the top chamber was pre‐coated with Matrigel. The lower chamber contained RPMI 1640 with 10% FBS. After 16 hours at 37C, the cells on the upper membrane surface were removed. The cells that migrated or invaded to the outside bottom surface were fixed with 4% paraformaldehyde, stained with 0.1% crystal violet, and counted. The assays were done in at least three independent experiments.

### 2.12 Gelatin zymography

Gelatin zymography was performed with an assay kit (XFBIO, XF‐P17750, Shanghai, China). The conditioned media from transduced THP‐1 cells were enriched by ultrafiltration (Amicon® Ultra 3K, Millipore) and run on 8% SDS‐polyacrylamide gels containing 0.1% gelatin. Recombinant human MMP‐2 protein (902‐MP‐010, R&D Systems) and the conditioned medium from cultured HeLa cells were used as positive controls. The gel was treated in the Buffer A from the kit for 24 hours. To activate MMPs, the gel was incubated in Buffer B from the kit at 37°C overnight with gentle shaking, followed by staining with 0.5% Coomassie Brilliant Blue R‐250, and subsequent destaining in 40% methanol and 10% acetic acid to visualize MMP cleavage bands. The images were acquired on an Amersham Imager 600 System (GE).

### 2.13 Xenograft in BALB/c nude mice

A xenograft model was conducted in athymic BALB/c (nu/nu) mice (Slake, China). The study was approved by the animal ethics committee of Soochow University. The mice (6‐week‐old males), housed in a specific‐pathogen–free facility with 12‐12‐hours light–dark cycles and free access to food and water, were divided into three groups (6 per group) by randomly picking a number assigned to each mouse. Investigators were not blinded to study groups. THP‐1–derived shNC, shH or shR cells with a green fluorescent protein tag (two cell clones for each cell type; 1 × 10^7^ cells in PBS) were injected subcutaneously into the lower right flank of the mice under sterile conditions. After 10 days, the tumor size was measured every two days using a slide gauge. The tumor volume was calculated using the following equation: tumor volume=tumor width^2 ^× tumor lenth/2. On day 36, the mice were anesthetized, examined with an in vivo imaging system (IVIS Lumina II) for tumor immages, and sacrificed by cervial dislocation. The tumors were dissected out, photographed, weighted and used for histological studies. No samples were excluded unless the animals died unexpectedly before the experiments were completed (one mouse in the shR1 group).

### 2.14 Immunohistochemistry

Tissues were fixed with 4% (v/v) formaldehyde, embedded in paraffin and sectioned as described previously.^37^ Immunohistochemistry was performed with primary antibodies against Ki‐67 and CD34 (MAB‐0672 and Kit‐0004, MXB Biotechnologies) and an HRP–conjugated secondary antibody (GeneTech, GK500710). In negative controls, the primary antibody was replaced with 5% BSA. The stained sections were examined under a light microscope (Leica DM2000) with a digital camera (Olympus, DP73).

### 2.15 TUNEL assay

A TUNEL assay kit (Roche) was used to stain apoptotic cells in tumor sections. The sections were treated with proteinase K for 30 minutes and incubated with an equilibration solution for 10 minutes and a terminal deoxynucleotidyl transferase reaction mixture for 1 hour to label DNA 3′‐hydroxyl termini. After washing with PBS, the sections were incubated with an HRP–conjugated antibody for 30 minutes, followed by staining with a DAB solution and counterstaining with hematoxylin. The sections were examined under a light microscope.

### 2.16 Statistical analysis

The sample size estimation was based on previous studies, pilot experiments and power calculation. Statistical analysis was done using SPSS 17.0 and Prism 7 software. Data equal variance and normality were verified using Levene's test and Kolmogorov‐Smirnov test, respectively. If the data passed the tests, Student's *t* test was used to compare two groups or one‐way analysis of variance (ANOVA) followed by Tukey test for comparisons among three or more groups. If the data did not pass the tests, Mann‐Whitney test was used for two independent sample comparisons, and Kruskal‐Wallis test and Mann‐Whitney test with Bonferroni correction were used for multiple comparisons. Correlations were analyzed with the Pearson method. *P* values < 0.05 are considered to be significant.

## 3 RESULTS

### 3.1 HAT‐L4 expression in hematological cancer cells

**Figure 1:**
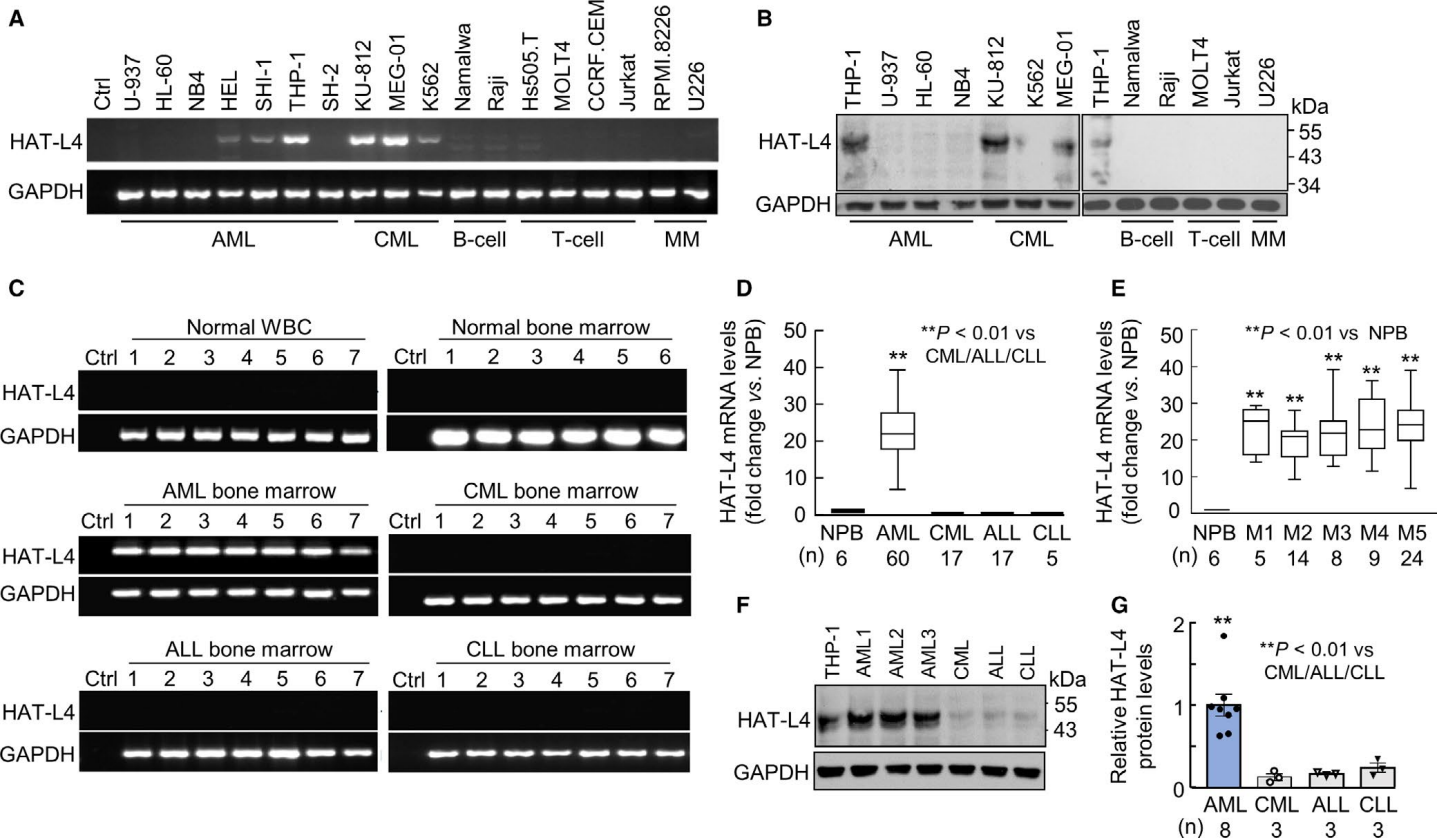
HAT‐L4 expression in leukemia cells. (A) RT‐PCR analysis of HAT‐L4 mRNA in human AML–, CML–, B‐cell–, T‐cell–, and multiple myeloma (MM)–derived cell lines. In controls (Ctrl), PCR was done without cDNA templates. (B) Western blotting of HAT‐L4 protein expression in human cancer cell lines. Data are representative of at least 3 independent experiments. (C) RT‐PCR analysis of HAT‐L4 mRNA expression in white blood cells (WBC) and bone marrow cells from normal individuals and bone marrow cells from AML, CML, ALL and CLL patients. The figures are representative. (D) qRT‐PCR analysis of HAT‐L4 mRNA levels in normal peripheral blood cells (NPB) and bone marrow cells from AML, CML, ALL and CLL patients. Sample numbers (n) in each group are indicated. Data were analyzed using Mann‐Whitney test. The confidence interval: AML (20.61, 24.28). (E) qRT‐PCR analysis of HAT‐L4 mRNA levels in subgroups (M1‐5) of AML patients. The data were analyzed by one‐way ANOVA. Confidence intervals: M1 (14.81, 30.84), M2 (16.36, 22.68), M3 (15.45, 29.15), M4 (17.90, 30.40) and M5 (20.37, 26.60). (F) Western blotting of HAT‐L4 protein expression in THP‐1 cells and bone marrow cells from AML, CML, ALL, and CLL patients. A representative blot is shown. (G) Protein bands on Western blots were scanned by densitometry. Quantitative data (mean ± SEM) were analyzed using one‐way ANOVA.

In RT‐PCR analysis of TTSP expression in human hematological cancer cells, we detected HAT‐L4 mRNA expression in AML–derived HEL, SHI‐1 and THP‐1 cells and CML–derived KU‐812, MEG‐01 and K562 cells (Figure 1A). Such expression was not detected in B‐cell–derived Namalwa and Raji cells, T‐cell–derived Hs 505.T, MOLT‐4, CCRF‐CEM and Jurkat cells or multiple myeloma–derived RPMI‐8226 and U226 cells (Figure 1A). Using western blotting, we detected HAT‐L4 protein of ~48 kDa in THP‐1, KU‐812 and MEG‐01 cells, but not in U‐937, HL‐60, NB4, K562, Namalwa, Raji, MOLT‐4, Jurkat and U226 cells (Figure 1B). The results suggested that HAT‐L4 may be ectopically expressed in myeloid leukemia.

### 3.2 HAT‐L4 expression in bone marrow cells from leukemia patients

To verify our findings, we examined HAT‐L4 expression in peripheral white blood cells (n = 7) and bone marrow cells (n = 6) from normal individuals, and bone marrow cells from AML (n = 60), CML (n = 17), ALL (n = 17), and CLL (n = 5) patients. Using RT‐PCR, we detected HAT‐L4 mRNA expression in all AML samples, but not in normal peripheral white blood cells (NPB), normal bone marrow cells, or CML, ALL and CLL bone marrow cells (Figure 1C). In qRT‐PCR, HAT‐L4 mRNA levels were ~22‐fold higher in AML bone marrow cells than those in NPB or bone marrow cells from other types of leukemia (Figure 1D). HAT‐L4 mRNA levels appeared similar in bone marrow cells from various AML FAB classes, including FAB M3 that is more typical for acute promyelocytic leukemia cells and behaves differently from the cells of the other FAB classes (Figure 1E).^32^ Western blotting confirmed high HAT‐L4 protein levels in AML bone marrow cells compared with those in CML, ALL and CLL bone marrow cells (Figure 1F,G).

### 3.3 HAT‐L4 expression on the cell surface

**Figure 2:**
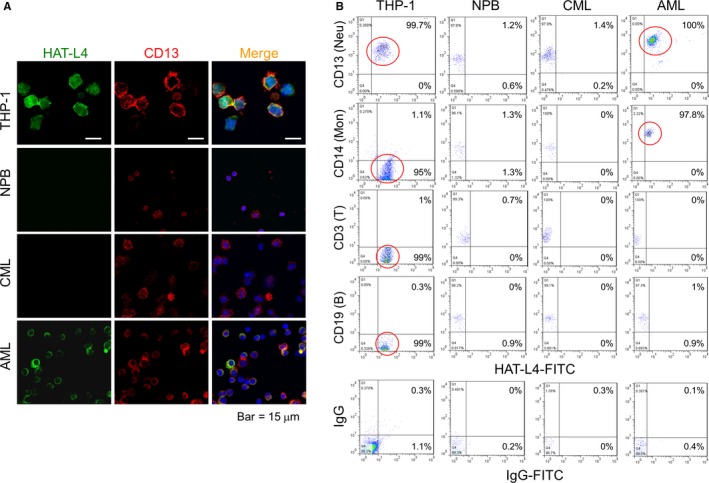
HAT‐L4 expression in THP‐1 and AML–derived bone marrow cells. (A) Immunostaining of HAT‐L4 (green) and CD13 (red) in THP‐1 cells, NPB, and CML‐ and AML–derived bone marrow cells, as analyzed using confocal microscopy. In merged panels, cell nuclei were stained in blue. (B) Flow cytometric analysis of HAT‐L4 expression on the surface of THP‐1, NPB and CML– and AML–derived bone marrow cells. Antibodies against cell markers, including CD13 for neutrophils (Neu), CD14 for monocytes (Mon), CD3 for T‐cells, and CD19 for B‐cells, were used. Isotype–matched IgG was included as a negative control. HAT‐L4‐positive populations are highlighted in red circles. Percentages of HAT‐L4‐positive cells in upper and lower left quadrants are indicated. Data are representative of at least three independent experiments

HAL‐L4 is a transmembrane protease.^29^ Using immunostaining, we detected HAT‐L4 protein on the surface of THP‐1 and AML bone marrow cells but not on NPB or CML bone marrow cells (Figure 2A). In controls, the myeloid antigen CD13 was detected on NPB, THP‐1, AML and CML cells. We also analyzed HAT‐L4 expression in AML bone marrow cell populations by flow cytometry. HAT‐L4 was positive in nearly all THP‐1 cells and AML–derived neutrophils and monocytes, but mostly negative in AML–derived lymphocytes and CML–derived neutrophils, monocytes and lymphocytes or NPB cells (Figure 2B), indicating that HAT‐L4 was ectopically expressed on the surface of AML–derived myeloid cells.

### 3.4 Association of HAT‐L4 expression with poor AML prognosis

**Figure 3:**
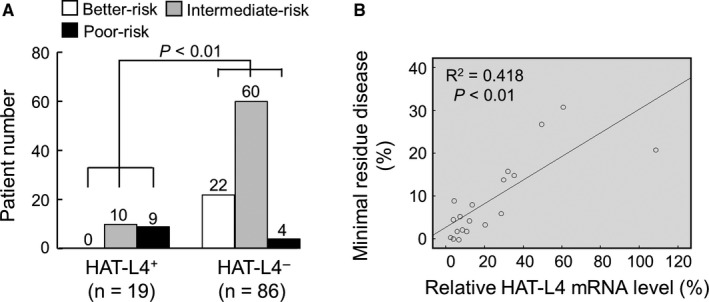
Correlation between HAT‐L4 expression and AML prognosis. (A) Prognostic risks were assessed in HAT‐L4^+^ (n = 19) and HAT‐L4^‐^ (n = 86) AML patients who underwent chemotherapy. Numbers of patients who were predicted to have better, intermediate and poor risks are shown on top of each bar. Statistical analysis was done by Chi‐square test. (B) In posttreatment AML patients who were HAT‐L4^+^ (n = 19), correlation between relative levels of HAT‐L4 mRNA expression and percentages of AML blasts in bone marrow cells was analyzed using the Pearson method

To understand the significance of HAT‐L4 expression in AML, we analyzed HAT‐L4 expression in bone marrow cells from 105 AML patients who underwent chemotherapy (7 days of cytarabine, 100‐200 mg/m^2^, infusion and 3 days of idarubicin 12 mg/m^2^). In qRT‐PCR analysis, 18% (19/105) of the posttreatment patients remained positive in HAL‐L4 mRNA expression in bone marrow cells (Table S1). Among them, 52.6% (10/19) were associated with intermediate prognostic risks and 47.4% (9/19) with poor prognostic risks (Figure 3A), as assessed by patients’ age, cytogenetics and gene mutations.^38^ In contrast, among the 86 AML patients who were HAT‐L4 negative in their bone marrow cells posttreatment (Table S1), 25.6% (22/86) were associated with better prognostic risks, 69.8% (60/86) with intermediate prognostic risks, and 4.6% (4/86) with poor prognostic risks (Figure 3A), indicating that posttreatment HAL‐4 expression in bone marrow cells was associated with poor prognosis. Among the 19 HAT‐L4–positive patients, HAT‐L4 mRNA levels correlated with the number of remaining AML cells in their bone marrows (Figure 3B). In correlation analysis, HAT‐L4 expression in AML bone marrow cells correlated with minimal residual disease (MRD) and poor prognostic risks, but not the gender, age or AML subtype in the posttreatment patients (Table S2).

### 3.5 Effects of HAT‐L4 silencing on THP‐1 cells

**Figure 4:**
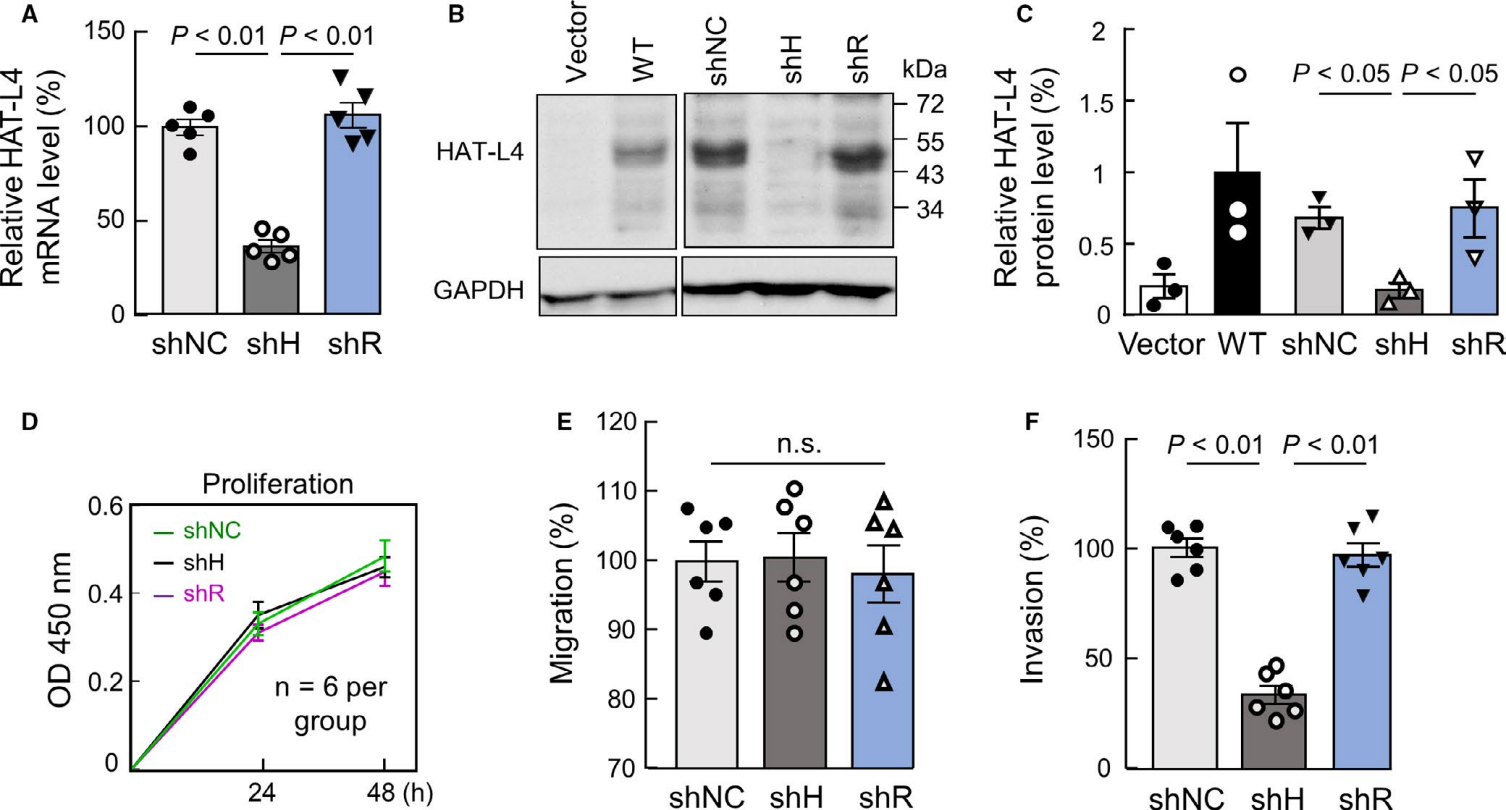
Effects of HAT‐L4 silencing on THP‐1 cell proliferation, migration and invasion. (A) qRT‐PCR analysis of HAT‐L4 mRNA levels in THP‐1–derived shNC (with scrambled shRNAs), shH (with HAT‐L4 targeting shRNAs) and shR (with HAT‐L4 resistant to targeting shRNAs) cells. (B) Western blotting of HAT‐L4 protein in shNC, shH and shR cells. CHO cells transfected with a vector (Vector) and human wild‐type (WT) HAT‐L4–expressing plasmid were used as negative and positive controls, respectively. (C) Protein bands on Western blots were quantified using densitometry. Data are shown as mean ± SEM. Results of cell proliferation (D), migration (E) and Matrigel invasion (F) are shown as mean ± SEM. Results were analyzed by one‐way ANOVA

To understand the role of HAT‐L4 expression in AML, we established THP‐1–derived shNC (transduced with scrambled shRNAs), shH (transduced with HAT‐L4‐targeting shRNAs) and shR (transduced with HAT‐L4–targeting shRNAs and HAT‐L4 cDNA with mutated targeting sites) cells. For the each cell type, two independent clones were used. In qRT‐PCR (Figure 4A) and Western blotting (Figure 4B,C), HAT‐L4 mRNA and protein levels were reduced markedly in shH cells compared with those in control shNC cells. In shR cells, which were resistant to HAT‐L4 silencing, HAT‐L4 mRNA and protein levels were comparable to those in shNC cells (Figure 4A‐C).

We examined the proliferation of the THP‐1–derived cells. In a CCK‐8 assay, shNC, shH and shR cells had similar proliferation rates (Figure 4D). In bromodeoxyuridine (BrdU) and propidium iodide (PI)–based cell cycle analysis, these cells also had similar cell cycle phases (data not shown), indicating that inhibition of HAT‐L4 expression in THP‐1 cells did not alter cell proliferation and cycles under our experimental conditions.

We next examined cell migration and invasion in transwell assays. Similar migration rates were observed for shNC, shH and shR cells to cross the fibronectin–coated polyester membrane (Figure 4E). In contrast, Matrigel invasion rate was reduced by 66.33 ± 12.07% in shH cells compared with that in control shNC cells (Figure 4F). Such a reduction was not observed in shR cells (93.70 ± 9.65% of shNC cells, *P* > 0.05). These results suggested that HAT‐L4 may promote THP‐1 cells to break down the extracellular matrix. In independent clones from shNC, shH and shR cells, similar results were confirmed on HAT‐L4 silencing, cell proliferation, migration and invasion (Figure S2).

### 3.6 HAT‐L4 and MMP activation

**Figure 5:**
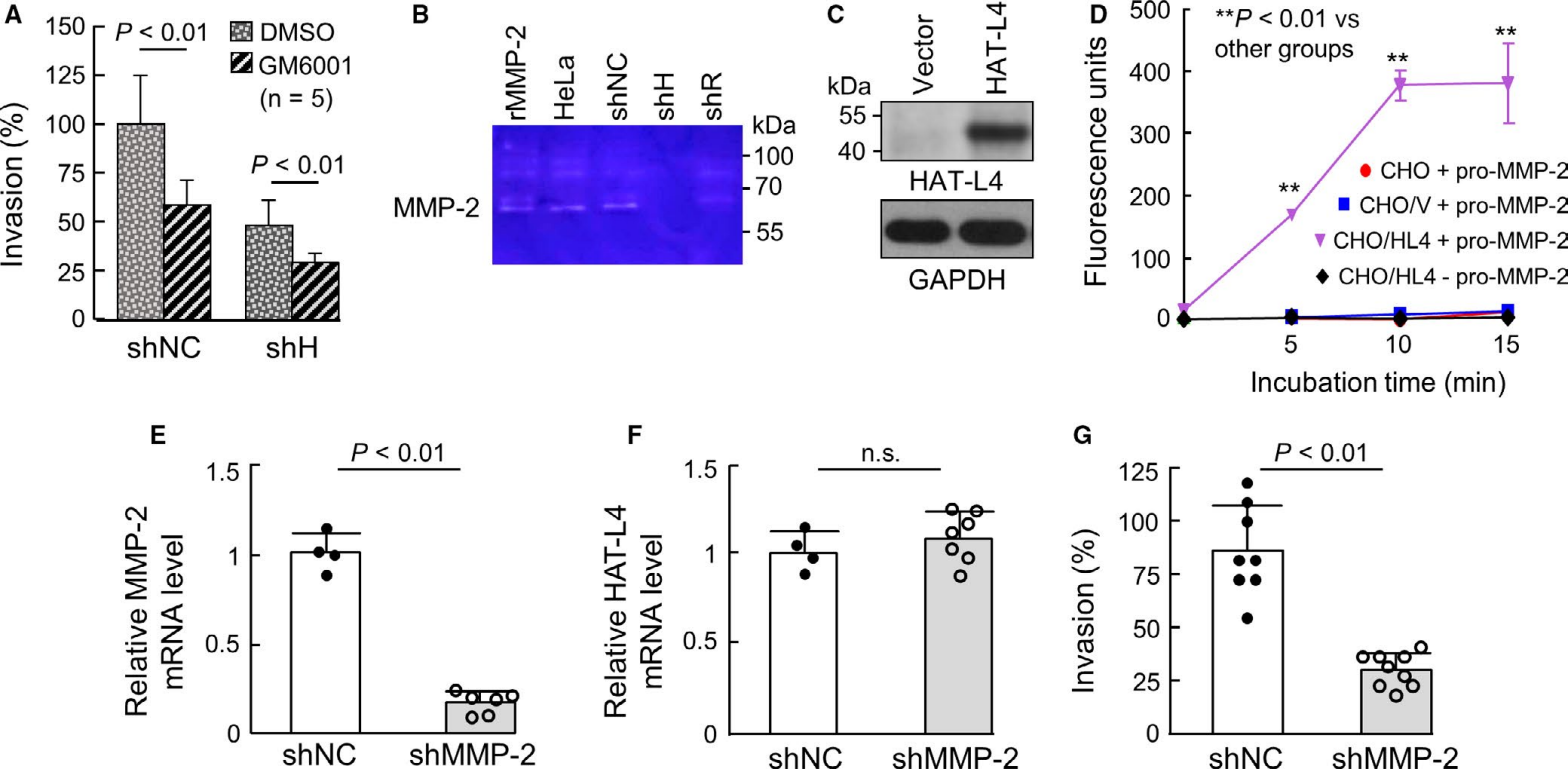
HAT‐L4 and MMP‐2 activation. (A) Effects of GM6001 on shNC and shH cells in Matrigel invasion. Results are mean ± SEM and analyzed using Student's *t* test. (B) Conditioned media from HeLa cells (positive control) and THP‐1–derived shNC, shH and shR cells were analyzed using zymography. Recombinant human MMP‐2 protein (rMMP‐2) was used as another positive control. (C) Western blotting of HAT‐L4 protein in CHO cells transfected with a control vector or HAT‐L4–expressing plasmid. GAPDH expression was used as a control. (D) MMP‐2 activity in the conditioned media from parental CHO (CHO), vector–transfected CHO (CHO/V) and HAT‐L4–expressing CHO (CHO/HL4) cells incubated with (+) or without (−) recombinant pro‐MMP‐2 was analyzed using a fluorogenic assay. Data were analyzed by one‐way ANOVA. (E) qPCR analysis of MMP‐2 mRNA levels in THP‐1 cells transduced with nontargeting shRNAs (shNC) or shRNAs targeting the MMP‐2 gene (shMMP‐2). (F) qPCR analysis of HAT‐L4 mRNA levels in THP‐1 cells transduced with shNC or shMMP‐2. (G) Matrigel invasion of THP‐1 cells transduced with shNC or shMMP‐2. Data in (A), (E), (F) and (G) were analyzed using Student's *t* test. Data in (D) were analyzed using one‐way ANOVA

MMPs are primary extracellular matrix‐degrading proteases. To test if HAT‐L4 promotes THP‐1 cell invasion by activating MMPs, we examined the effect of GM6001, a broad MMP inhibitor, on Matrigel invasion of shNC and shH cells. In the presence of GM6001, Matrigel invasion was inhibited in shNC and, to a less degree, shH cells (Figure 5A), suggesting that HAT‐L4 may promote THP‐1 cell invasion, in part, via an MMP–dependent mechanism.

We next did zymography to examine MMP activity in the conditioned media from the THP‐1–derived cells. A ~62‐kDa band was detected in samples from HeLa cells (positive control)^39^ as well as shNC and shR cells, but not shH cells (Figure 5B). As a control, recombinant human MMP‐2, a primary member in the MMP family, migrated at a similar position (Figure 5B). To verify these results, we expressed HAT‐L4 in CHO cells. Western blotting confirmed HAT‐L4 protein in CHO cells transfected with HAT‐L4–expressing plasmid but not in vector–transfected cells (Figure 5C). In a fluorogenic assay, MMP‐2 activity was detected when recombinant pro‐MMP‐2 was incubated with the conditioned medium from HAT‐L4–expressing CHO cells, but not the medium from the vector–transfected or parental CHO cells. In another control, no activity was detected in the conditioned medium with HAT‐L4 but no pro‐MMP‐2 (Figure 5D). We next knocked down MMP‐2 expression in THP‐1 cells by shRNAs. In the cells transduced with shRNAs targeting MMP‐2 (shMMP‐2), MMP‐2, but not HAT‐L4, mRNA levels were markedly reduced (Figure 5E,F). Such an effect was not observed in THP‐1 cells transduced with nontargeting shRNAs (shNC). In the Matrigel invasion assay, reduced invasion was found in shMMP‐2–treated THP‐1 cells, compared with that in shNC–treated THP‐1 cells (Figure 5G). These results indicate that HAT‐L4 may activate MMP‐2, which in turn degrades extracellular matrix to promote THP‐1 cell invasion in Matrigels.

### 3.7 Effects of HAT‐L4 silencing on THP‐1–derived tumors in mice

**Figure 6:**
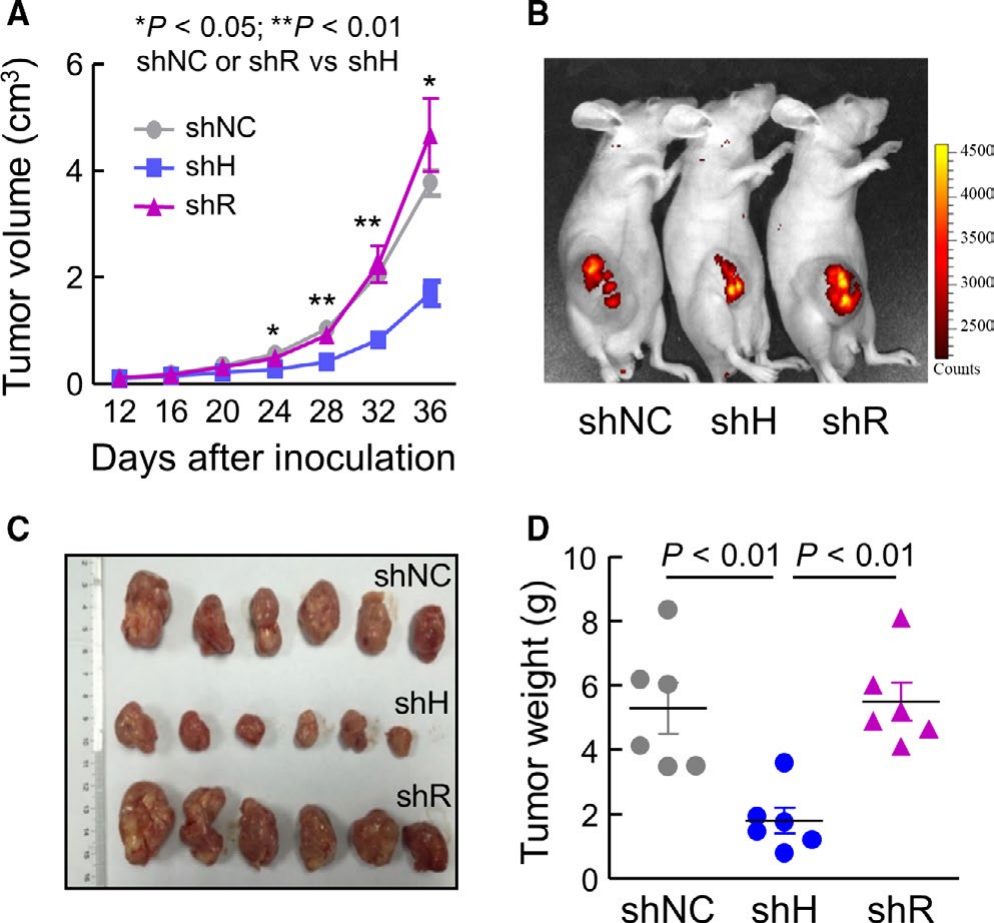
Effects of HAT‐L4 silencing on the growth of THP‐1 cell–derived tumors in mice. (A) Tumor volumes in athymic nude mice inoculated with shNC, shH and shR cells. n = 6 per group. Data were analyzed using one‐way ANOVA. (B) Live‐imaging of shNC, shH and shR cell–derived tumors in representative mice on day 36 postinoculation. (C) Images of dissected tumors from shNC, shH and shR cells. (D) Tumors from shNC, shH and shR cells were weighed. Data were analyzed using one‐way ANOVA

To test if HAT‐L4 expression promotes tumor growth, we established a xenograft tumor model in mice using THP‐1–derived shNC, shH and shR cells. For each cell line, two independent clones were used. Compared with shNC or shR cell–derived tumors, shH cell–derived tumors grew slower in vivo, as indicated by smaller tumor volumes (Figure 6A). On postinoculation day 36, live‐imaging showed weaker fluorescent signals in mice with shH cell–derived tumors compared to those with shNC or shR cell–derived tumors (Figure 6B). Consistently, shH cell–derived tumors, when dissected out, were smaller (Figure 6C) and lighter (Figure 6D) compared with shNC or shR cell–derived tumors.

**Figure 7:**
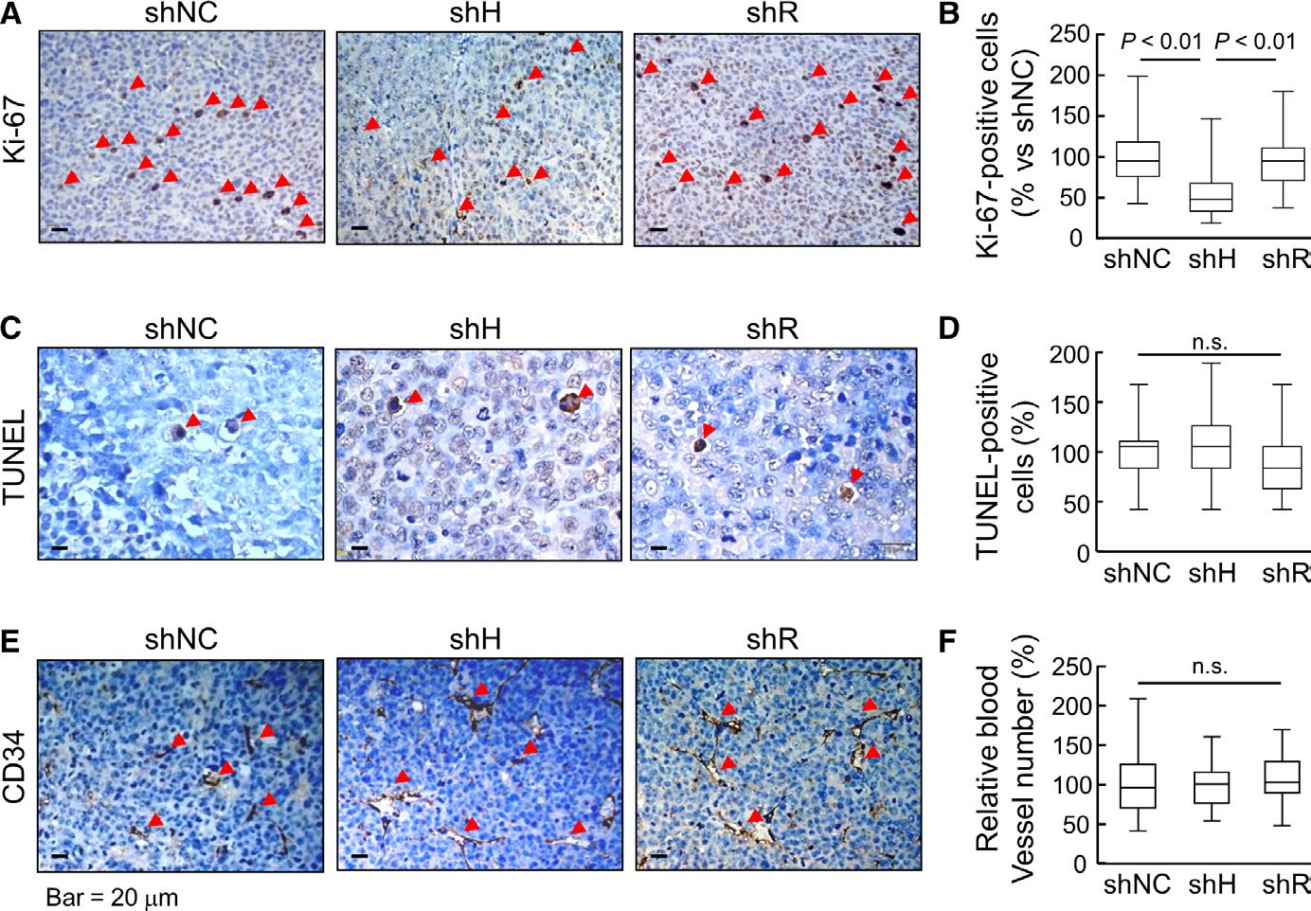
Cell proliferation, apoptosis and angiogenesis in tumors. Ki‐67 (A), TUNEL (C) and CD34 (E) staining were done in shNC, shH and shR cell–derived tumor sections. For each group, 90 randomly selected fields in 18 sections from 6 mice were examined under a light microscope. Ki‐67–positive cells in (A), TUNEL–positive cells in (C) and CD34–positive cells in (E) are indicated by red arrowheads. Quantitative data in (B), (D) and (F) were analyzed by Kruskal‐Wallis test and Mann‐Whitney test with Bonferroni correction. Confidence intervals in (B): shNC (92.71, 107.29), shH (47.93, 59.54) and shR (89.46, 103.60); (D): shNC (94.08, 105.92), shH (95.19, 105.56) and shR (87.48, 100.37); and (F) shNC (92.88, 107.12), shH (93.20, 104.29) and shR (101.57, 113.08)

In tissue sections, numbers of Ki‐67–positive cells, a marker for proliferating cells, were less in shH cell–derived tumors compared with those in shNC and shR cell–derived tumors (Figure 7A,B). In contrast, TUNEL–positive cells were similar in all tumors (Figure 7C,D). CD34 staining, a vascular marker, was also comparable in shNC, shH and shR cell–derived tumors (Figure 7E,F). In independent clones from the THP‐1–derived tumors, similar results were confirmed in tumor growth (Supporting Information Figure S3) and Ki‐67‐, TUNEL‐ and CD34‐staining (Figure S4). These results indicated that silencing HAT‐L4 expression inhibited THP‐1 cell–derived tumor proliferation and growth in the mouse xenograft model.

## 4 DISCUSSION

AML is a major malignant hematological disease. In this study, we identified ectopic expression of HAT‐L4, an epithelial transmembrane serine protease, in human myeloid leukemia cell lines and bone marrow cells from AML, but not CML, ALL and CLL, patients. Immunostaining and flow cytometry detected HAT‐L4 expression on the surface of AML–derived neutrophils and monocytes. In AML patients who underwent chemotherapy, persistent HAT‐L4 expression in bone marrow cells correlated with MRD and poor prognosis. We also analyzed public gene expression databases^40^ and found similar results of high levels of HAT‐L4 expression in AML cells. A summary of our findings is listed in Table S3. The results suggest that the HAT‐L4 expression is an indicator of AML blasts in the bone marrow.

Normally, human HAT‐L4 is expressed in epithelial cells and exocrine glands in the skin, esophagus, trachea, testis and placenta.^28^, ^29^ By RT‐PCR, we did not detect HAT‐L4 mRNA expression in peripheral white blood cells or bone marrow cells from normal individuals. Consistently, HAT‐L4‐deficient mice had normal blood cell counts, including red blood cells, white blood cells and platelets.^29^ The mechanism underlying the ectopic HAT‐L4 expression in AML is unclear. Among TTSPs overexpressed in cancer, TMPRSS2 expression in prostate cancer is regulated by an androgen response element in the gene promotor.^22^, ^41^ Moreover, gene translocations have been identified between the *TMPRSS2* promotor and members of the E26 transformation‐specific transcription factor family, which disrupts normal androgen receptor signaling and activates tumor–promoting epigenetic programs.^42^, ^43^, ^44^ The human *TMPRSS11F* gene, encoding HAT‐L4, is located on chromosome 4 at the position 68053198‐68129869. By analyzing the NCBI databases, we did not find any AML–associated chromosomal translocations in this region. Among 105 AML patients in our study, none of them had chromosomal translocations near the *TMPRSS11F* gene (Table S1). Consistently, there were no correlations between cytogenetics and HAT‐L4 expression in AML cells in our study. Additional studies are required to determine the mechanisms underlying the ectopic HAT‐L4 expression in AML cells.

To understand the significance of HAT‐L4 expression in AML cell biology, we tested the effects of HAT‐L4 silencing on THP‐1 cells. Our results show that HAT‐L4 down‐regulation did not inhibit THP‐1 cell proliferation or migration in culture but blocked the invasion of these cells in Matrigels. In experiments with the MMP inhibitor GM6001, zymography and fluorogenic substrate assays, we found that HAT‐L4 may promote THP‐1 cell invasion by activating MMP‐2, a major MMP that degrades extracellular matrixes, contributing to cancer invasion and metastasis.^45^ In AML, breaking down the extracellular matrix is critical for leukemia blasts to escape the bone marrow and invade in extramedullary sites such as the liver, spleen and skin.^46^ High levels of MMP‐2 expression have been reported in AML.^47^, ^48^ Moreover, MMP‐2 overexpression was associated with enhanced invasiveness of drug–resistant AML cells and poor clinical outcomes.^47^, ^49^ Previously,^50^ Zhou *et al* reported MMP‐2 and MMP‐9 expression in THP‐1 cells, which was enhanced by phorbol myristate acetate. The up‐regulation was much greater for MMP‐9 than MMP‐2.^50^ In our experiments, we used untreated THP‐1 cells and found MMP‐2 as a major band in THP‐1 cell–derived media by zymography. We could not exclude the possibility that MMP‐9 might be present at lower levels in the media. By knocking down MMP‐2 expression, we found markedly reduced THP‐1 invasion in Matrigels. These data support the idea that in AML HAT‐L4 may function, at least in part, by enhancing MMP‐2 activation, which in turn promotes leukemia blast invasion and progression.

In agreement with the findings in vitro, the THP‐1–derived cells, in which HAT‐L4 expression was down‐regulated by shRNAs, grew slower than the control cells in athymic nude mice. Analysis of xenograft tumor sections by Ki‐67‐, CD34‐ and TUNEL‐staining indicated that blocking HAT‐L4 expression in THP‐1 cell–derived tumors inhibited cell proliferation but not angiogenesis or apoptosis. The mechanism underlying the inhibitory effect on THP‐1 cell proliferation in vivo is unclear. In CCK‐8 assay, blocking HAT‐L4 expression did not inhibit THP‐1 cell proliferation in vitro. Possibly, the increased cell proliferation in HAT‐L4–expressing THP‐1 cell–derived tumors in vivo is a consequence of enhanced invasiveness of these cells in tumor tissues. For example, increased extracellular matrix degradation may create a tumor microenvironment that favors cancer cell growth.^51^, ^52^ Alternatively, the inhibitory effect of blocking HAT‐L4 expression on THP‐1 cell proliferation in the xenograft model was mediated by HAT‐L4 substrate(s) that were absent in our cell culture. Previously, matriptase, an epithelial TTSP, was shown to activate growth factors, such as hepatocyte growth factor,^53^ macrophage stimulating protein 1,^54^ and members of the platelet‐derived growth factor family,^55^ which promote cancer progression by enhancing c‐Met–, PAR‐2–, RON– and PDGF receptor–mediated pathways. Further in vitro and in vivo studies are required to test if HAT‐L4 can directly cleave and activate growth factors that are associated with AML cell proliferation and progression. Findings from THP‐1 cells should also be verified in other AML–derived cells.

In summary, HAT‐L4 is a transmembrane protease expressed in epithelial cells and exocrine glands. Here we report an unexpected finding of ectopic HAT‐L4 expression in neutrophils and monocytes from AML patients. In AML patients who underwent chemotherapy, persistent HAT‐L4 expression in bone marrow cells indicated remaining leukemia blasts and poor prognosis. In cell culture, blocking HAT‐L4 expression in THP‐1 cells inhibited MMP‐2 activation and Matrigel invasion. In a mouse xenograft model, blocking HAT‐L4 expression decreased the proliferation and growth of THP‐1 cell–derived tumors. Together, our results indicate that ectopic HAT‐L4 expression is a pathological mechanism in AML and that HAT‐L4 may be used as a cell surface marker for AML blast detection and targeting. Our findings should encourage additional studies to examine if HAT‐L4 is ectopically expressed in additional abnormal blood cells, such as myelodysplastic syndrome and other preleukemia cells.

## CONFLICT OF INTERESTS

The authors declare that they have no competing interests.
