# Correction to “Ectopic expression of human airway trypsin‐like protease 4 in acute myeloid leukemia promotes cancer cell invasion and tumor growth”

**DOI:** 10.1002/cam4.70700

**Published:** 2025-03-02

Yan R, Liu M, Hu Y, et al. Ectopic expression of human airway trypsin‐like protease 4 in acute myeloid leukemia promotes cancer cell invasion and tumor growth. *Cancer Med.* 2019;8:2348–2359, https://doi.org/10.1002/cam4.2074.

It was brought to our attention that errors occurred in Figures 3B and 7E. In Figure 3B, the lines and circles were shifted during figure preparation, leaving an impression that two low data points were in negative values. The correlation coefficient was 0.804, as listed in Table S2. The *R* ^2^ value was miscalculated and should be 0.646. In Figure 7E, two images taken from the same tissue section of the shR group were mistakenly used in the shH and shR groups. Corrected Figures 3B and 7E are included below. The conclusions of the paper remain unchanged.

We apologize for the errors.

**Figure:**
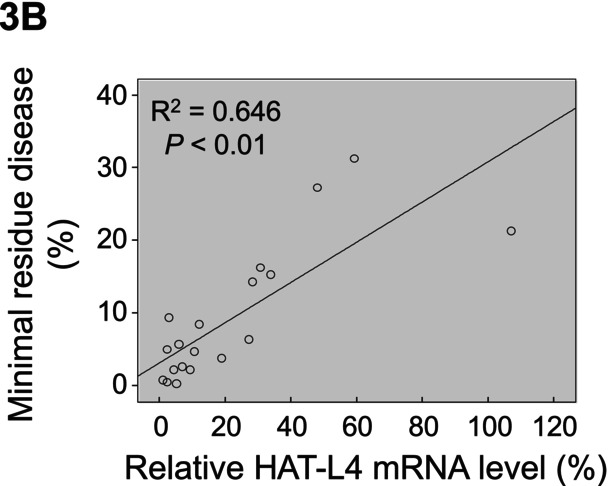
3B

**Figure:**
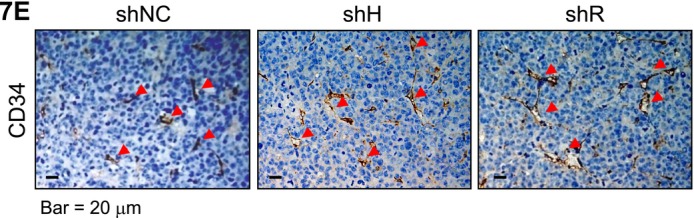
7E

