# Supplementary material for: Ectopic expression of human airway trypsin‐like protease 4 in acute myeloid leukemia promotes cancer cell invasion and tumor growth
Source: Cancer Med. 2019 Mar 7;8(5):2348–59. doi: 10.1002/cam4.2074 (PMC6537003; doi:10.1002/cam4.2074)
Supplement: Supplementary file 1 [file CAM4-8-2348-s001.pdf]

Supporting Information

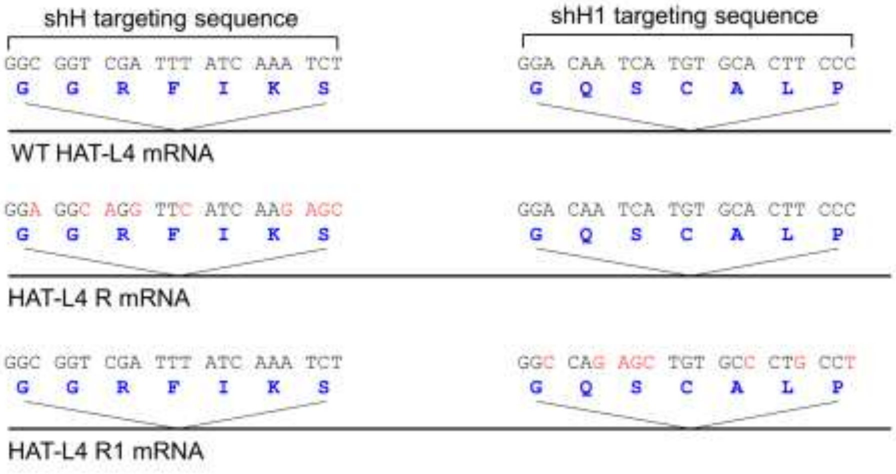

Fig. S1. Illustration of HAT-L4 silencing strategy. Sequences of shRNA-targeting sites in human HAT-L4 cDNA are shown. Mutant HAT-L4 cDNAs were made by site-directed mutagenesis to alter the shRNA-targeting sites (base pairs in red) without changing encoded amino acids (in blue).

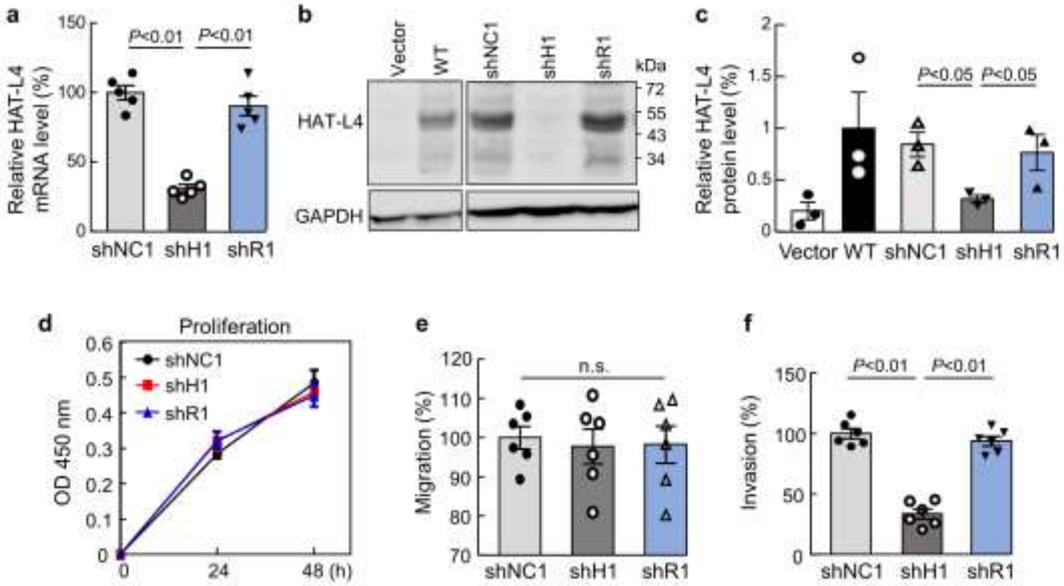

Fig. S2. Effects of HAT-L4 silencing on THP-1 cell proliferation, migration and invasion, which were tested in independent cell lines. (**a**) qRT-PCR analysis of HAT-L4 mRNA levels in THP-1-derived shNC1 (with scrambled shRNAs), shH1 (with HAT-L4 targeting shRNAs) and shR1 (with HAT-L4 resistant to targeting shRNAs) cells. (**b**) Western blotting of HAT-L4 protein in shNC1, shH1 and shR1 cells. Cell lysates were run on the same Western blot shown in Fig. 4b, which had the same negative and positive controls (CHO cells transfected with a vector (Vector) and wild-type (WT) HAT-L4-expressing plasmid) (left panels). (**c**) Protein bands on Western blots were quantified by densitometry. Data are shown as mean ± SEM. Results of cell proliferation (**d**), migration (**e**) and Matrigel invasion (**f**) are shown as mean ± SEM. Results in **a** and **c**-**f** were analyzed by one-way ANOVA.

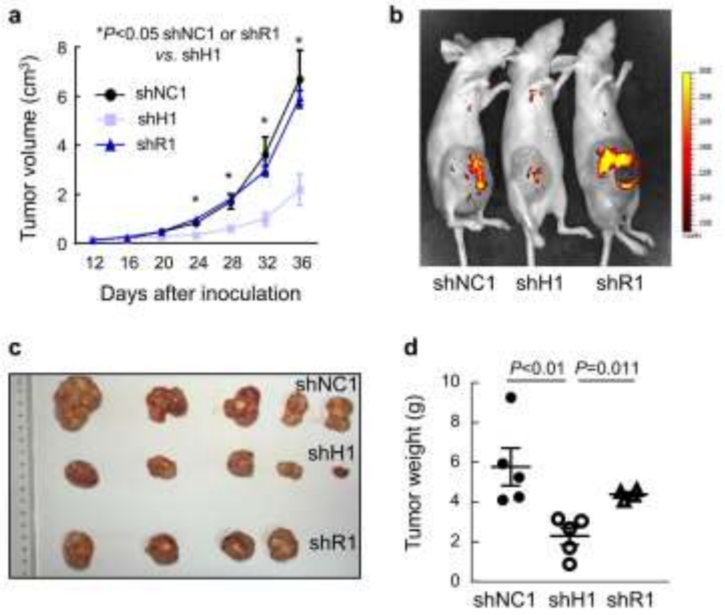

Fig. S3. Effects of HAT-L4 silencing on the growth of THP-1 cell-derived tumors in mice, which were tested in independent cell lines. (**a**) Tumor volumes in athymic nude mice inoculated with shNC1 (n=5), shH1 (n=5) and shR1 (n=4) cells. Data were analyzed by one-way ANOVA. (**b**) Live-imaging of shNC1, shH1 and shR1 cell-derived tumors in representative mice on day 36 post-inoculation. (**c**) Images of dissected tumors from shNC1, shH1 and shR1 cells. (**d**) Tumors from shNC1, shH1 and shR1 cells were weighed. Data were analyzed by one-way ANOVA.

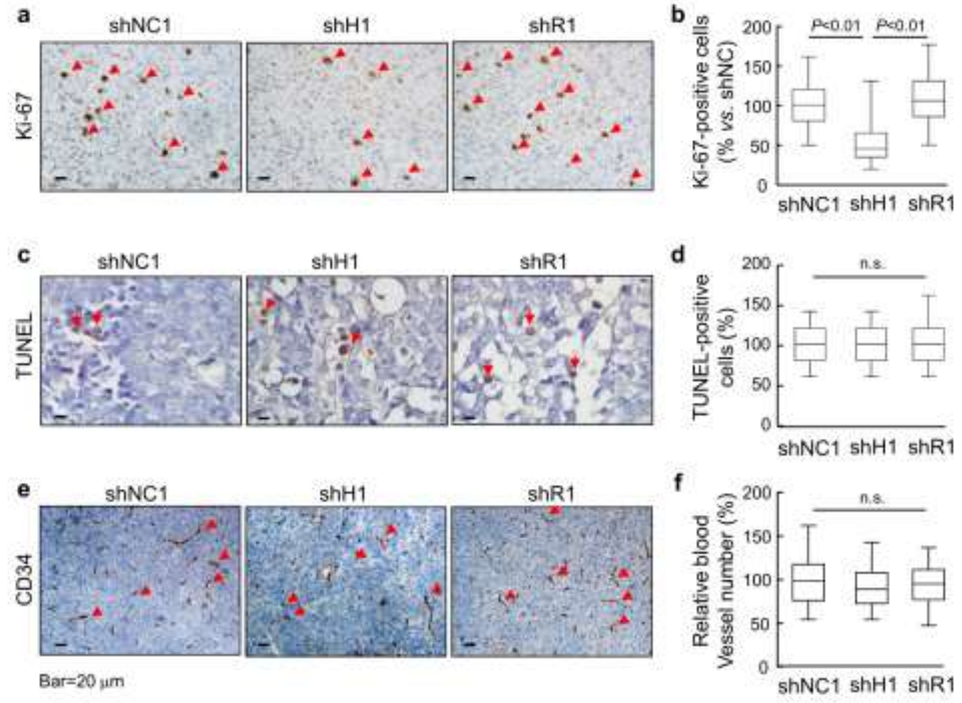

Fig. S4. Cell proliferation, apoptosis and angiogenesis in tumors, which were tested in independent cell lines. Ki-67 (**a**), TUNEL (**c**) and CD34 (**e**) staining were done in sections from shNC1 (n=5), shH1 (n=5) and shR1 (n=4) cell-derived tumors. For each group, 15 randomly selected fields in 3 sections from each tumor were examined under a light microscope. Ki-67-positive cells in (**a**), TUNEL-positive cells in (**c**), and CD34-positive cells in (**e**) are indicated by red arrowheads. Quantitative data in (**b**), (**d**) and (**f**) were analyzed by Kruskal-Wallis test and Mann-Whitney test with Bonferroni correction.

Table S1. Characteristics of 105 AML patients after treatment

patient	Karyotype	Molecular genetic aberrations	FAB	HAT-L4
1	46,XX[20]	N	M2	+
2[a]	46,XY[20]	NPM1 A, DNMT3A (R882H)	M5	+
3	46,XX[20]	N	M4	+
4	46,XY[20]	FLT3-ITD, CEBPA bZIP	M2	+
5	46,XY,t(11;19)(q23;p13)[10]	CEBPA	M1	+
6	46,XX,del(8)(q22)[7]/46,idem ,+ace1-2[3]	N	M2a	+
7	46,XX[20]	N	M4a	+
8[a]	46,XY[20]	FLT3-ITD	M4	+
9	46,XY[20]	NPM1A	M2	+
10	46,XX[20]	N	M2a	+
11	46,XY[20]	WT1	M5	+
12	46,XX[20]	WT1	M5	+
13	46,XX[20]	WT1	M2a	+
14[a]	46,XX[20]	FLT3-ITD, DNMT3A	M5	+
15	46,XX[20]	N	M4	+
16	46,XY[20]	N	M4	+
17	46,XX[20]	FLT3-ITD, DNMT3A (R882H)	M5	+
18	46,XX[20]	N	M5	+
19	46,XX[20]	FLT3-ITD, DNMT3A	M5	+
20	46,XY[20]	N	M1	-
21	47,XX,+11[5]/47,XX,+8[3]/46 , XX[2]	N	M5	-
22	46,XY[20]	N	M3	-
23	46,XX[20]	G250E, F359C, Ph+	M2a	-
24	46,XYt(11;19)(q23;p13)[8]/47 , idem,+8[5]/46,xy[7]	N	M5	-
25	45,X,- Y,t(8;21)(q22;q22)[8]/46,XY[2]	AML1-ETO	M2	-
26	46,XY[20]	N	M5	-
27	46,XY[20]	N	M2	-
28	46,XY[20]	CEBPA	M5	-
29	46,XY[20]	CEBPA TAD1, CEBPA bZIP	M1	-
30	46,XY[20]	CEBPA	M1	-
31	46,XX,t(8;21)(q22;q22)	AML1-ETO	M4	-
32	46,XY[20]	AML1-ETO	M2a	-
33	46,XX[20]	c-KIT17, CBFβ/MYH11	M4Eo	-
34	46,XX[20]	NPM1	M2a	-
35	46,XX[20]	N	M2a	-
36	46,XY,t(15;17)(q22;q12)	N	M3b	-
37	46,XY[20]	FLT3-TKD, D835Y, NPM1, DNMT3A	M5	-
38	46,XY[20]	NPM1A	M1	-
39	46,XX,t(15;17)(q22;12)	Ph+	M3a	-
40	46,XY[20]	NPM1A, DNMT3A (R882H)	M2	-
41	46,XY[20]	N	M2	-
42	46,XX[20]	N	M2	-
43	46,XX,t(6;9)(p23;q34)[5]/46,	DEK-CAN	M4	-

	XX[5]			
44	46,XX[20]	N	M4E0	-
45	45,XY,t(3;3)(q21;q26),-7[7]/46,XY[3]	N	M1	-
46	46,XY[20]	NPM1	M5	-
47	46,XY[20]	NPM1	M2	-
48	46,XX[20]	N	M1	--
49	46,XX[20]	NPM1, DNMT3A	M4EO	-
50	46,XY[20]	N	M3	-
51	Inv(16)[7]/47,idem,+22[3]	CBFβ/MYH11	M4	-
52	46,XY[20]	N	M4	-
53	46,XY[20]	Ph+	M3	-
54	46,XY[20]	Ph+	M3	-
55	46,XY[20]	N	M4	-
56	46,XY[20]	N	M2	-
57	46,XX[20]	FLT3-ITD	M5	-
58	45,XY,-7[20]	N	M5b	-
59	46,XX,del(9)(q12;q32)[5]/46,X X[10]	N	M2	-
60	46,XY,t(8;21)(q22;q22)	AML1-ETO	M2	-
61	46,XY[20]	N	M3	-
62	46,XY[20]	N	M4	-
63	46,XY[20]	FLT3-TKD, Ph+	M3b	-
64	45,XY,-7[2]/46,XY[2]	N	M1	-
65	46,XY[20]	CBFβ/MYH11	M2	-
66	46,XY[20]	AML1-ETO	M2	-
67	46,XX[20]	N	M2a	-
68	46,XX[20]	NPM1	M1	-
69	48,XY,+8,+8,t(9;11)(q11;q23)	N	M5	-
70	46,XX[20]	AML1-ETO	M2	-
71	46,XY[20]	N	M2	-
72	46,XY[20]	DNMT3A	M4	-
73	46,XX[20]	CEBPA	M1	-
74	46,XX[20]	N	M5	-
75	46,XY[20]	N	M5b	-
76	46,XY[20]	FLT3-TKD	M2	-
77	45,X,-Y,t(8;21)(q22;q22),11q-[5]46/46,XY[5]	AML1-ETO	M2	-
78	47,XY,+8	N	M5	-
79	46,XY,t(8;21)(q22;q22),9q-[10]	AML1-ETO	M2	-
80	46,XY[20]	N	M1	-
81	46,XX[20]	CEBPA	M1	-
82	46,XY[20]	FLT3-ITD	M1	-
83	46,XY[20]	AML1-ETO	M2	-
84	46,XX[20]	CBFβ/MYH11	M4	-
85	46,XY[20]	CEBPA	M2	-
86	46,XY,t(8;21)(q22;q22)[4]/46,XY[6]	AML1-ETO	M2a	-
87	46,XY[20]	N	M1	-
88	46,XY[20]	N	M4	-
89	46,XY[20]	CEBPA TAD1, CEBPA bZIP	M5	-

90	46,XY[20]	DNMT3A	M4	-
91	46,XY[20]	CEBPA	M2	-
92	45,X,-Y,t(8;21)	AML1-ETO	M2	-
93	46,XX,t(8;21)(q22;q22)	AML1-ETO	M4	-
94	46,XY[20]	N	M1	-
95	46,XY[20]	N	M4	-
96	46,XY,6q-[3]/46,idem,1q-[2]/46,XX[5]	N	M4	-
97	46,XX[20]	N	M1	-
98	46,XY[20]	N	M5	-
99	46,XY[20]	NPM1	M5	-
100	46,XX[20]	N	M3	-
101	46,XY[20]	N	M3	-
102	46,XY[20]	N	M1	-
103	46,XY[20]	N	M3	-
104	45,X,-Y,t(8;21)(q22;q22)[10]	c-KIT	M2	-
105	46,XX[20]	N	M5b	-

N：normal；[a]：relapse

Table S2. Correlations between HAT-L4 expression in AML bone marrow cells and clinical parameters

Variable	Correlation coefficient	*P* value
Subtype	0.29	0.23
Sex	0.10	0.70
Age (years)	0.19	0.44
Minimal residue disease	0.80	<0.01
Poor prognostic risk	0.46	0.04

Table S3. A summary of the main findings

Findings	Methods
HAT-L4 expression was detected in AML-derived cell lines.	RT-PCR; Western
HAT-L4 was ectopically expressed in AML cells, but not in CML ALL and CLL cells.	RT-PCR; qPCR; Western
HAT-L4 was expressed on the surface of THP-1 and AML cells.	Flow cytometry
In AML patients, HAT-L4 expression correlated with MRD and poor prognosis.	qPCR; clinical data analysis
In THP-1 cells, down-regulation of HAT-L4 expression inhibited cell invasion in Matrigels.	shRNA knock-down; mRNA and protein analysis; in vitro assays
Identify a role of MMP-2 in HAT-L4-dependent cell invasion.	GM6001 inhibition; Zymography; Western; Substrate assay; MMP-2 knockdown cells
Down-regulation of HAT-L4 expression inhibited the growth of THP-1 cell-derived tumors in a nude mouse model	Measurements of tumor volume and weight; histological analysis and immune staining of tumor tissue sections
